# Pathway dissection, regulation, engineering and application: lessons learned from biobutanol production by solventogenic clostridia

**DOI:** 10.1186/s13068-020-01674-3

**Published:** 2020-03-06

**Authors:** Shubo Li, Li Huang, Chengzhu Ke, Zongwen Pang, Liming Liu

**Affiliations:** 1 College of Light Industry and Food Engineering, Guangxi University, Nanning, 530004 China; 2 College of Life Science and Technology, Guangxi University, Nanning, 530005 China; 3 State Key Laboratory of Food Science and Technology, Jiangnan University, Wuxi, 214122 China

**Keywords:** Acetone–butanol–ethanol fermentation, Solventogenic clostridia, Metabolic characteristics, Metabolic engineering, Strain improvement, Process optimization

## Abstract

The global energy crisis and limited supply of petroleum fuels have rekindled the interest in utilizing a sustainable biomass to produce biofuel. Butanol, an advanced biofuel, is a superior renewable resource as it has a high energy content and is less hygroscopic than other candidates. At present, the biobutanol route, employing acetone–butanol–ethanol (ABE) fermentation in *Clostridium* species, is not economically competitive due to the high cost of feedstocks, low butanol titer, and product inhibition. Based on an analysis of the physiological characteristics of solventogenic clostridia, current advances that enhance ABE fermentation from strain improvement to product separation were systematically reviewed, focusing on: (1) elucidating the metabolic pathway and regulation mechanism of butanol synthesis; (2) enhancing cellular performance and robustness through metabolic engineering, and (3) optimizing the process of ABE fermentation. Finally, perspectives on engineering and exploiting clostridia as cell factories to efficiently produce various chemicals and materials are also discussed.

## Background

Due to the limited supply of petroleum oil, mounting environmental concerns, and an awareness of the energy crisis, it has become necessary to investigate a renewable biofuel as a substitute for oil [1, 2]. Butanol, a four-carbon primary alcohol, is appealing commercially not only as an important bulk chemical, but also as a promising biofuel because of its superior characteristics [3–5]. However, commercial butanol is currently derived via a petrochemical route based on propylene oxo synthesis, which is extremely sensitive to the price of crude oil [3]. Therefore, as the most viable route of biobutanol production, the acetone–butanol–ethanol (ABE) fermentation using *Clostridium* species has been attracting increasing interest in both academia and industry [6, 7], and several retrofitted and new industrial plants are under construction or even operating globally [8].

Once one of the largest fermentation industries, clostridia-based ABE fermentation [producing acetone, butanol, and ethanol at the ratio of 3:6:1 (mass ratio)] was first reported by Louis Pasteur in 1861, and developed to an industrial production level by Chaim Weizmann in 1912 [9]. Unfortunately, despite the substantial improvements that have been obtained over the past 100 years, several drawbacks still severely detract from the economic feasibility of ABE fermentation, including the: (1) high cost of substrate and substrate inhibition; (2) sluggish growth and low cell density; (3) low butanol concentration (< 20 g/L), yield (< 0.33 g/g), and productivity (< 0.5 g/L/h); (4) undesirable butanol selectivity; (5) extra energy consumption for butanol purification; (6) few tools appropriate for genetic engineering, and (7) poor understanding of the physiological characteristics of clostridia [1, 10, 11] (Table 1).

**Table 1:** Major bottlenecks and possible solutions for ABE fermentation [85]

Challenges	Influences	Possible solutions
High feedstock cost	Substrates are expensive and compete with nutrition (food versus fuel debate)	(i) Construction of novel host or introduction of novel metabolic pathway to utilize cheaper substrates and even carbon dioxide (ii) Development of physical/chemical pretreatment processes that do not require expensive enzymatic treatment
Low production, yield, and productivity of butanol	Increasing feedstock costs, recovery costs, water usage, energy-intensive process, and the cost of effluent treatment	(i) Develop strains with improved solvent production and/or develop methods for in situ product removal to alleviate product inhibition (ii) Develop continuous fermentation processes to increase volumetric productivity (iii) Develop low-energy methods for solvent recovery and purification
Poor genetic engineering tools	Metabolic engineering was inhibited by the low frequencies of transformation and recombination	(i) Develop more plasmid vectors and more efficient transformation methods (ii) Develop more efficient gene knockout methods
Poor understanding of physiology	Lack of knowledge of the entire genome and specific genes in the metabolic pathway that could further improve cellular performance	(i) Gain a detailed characterization and understanding of acidogenic and solventogenic phases and phase transition (ii) Systematically develop an understanding of the complex regulatory circuits and their interactions with metabolism
Bacteriophage contamination	(1) Decreasing fermentation rate, (2) change in bacterial cell population and morphology, (3) high levels of unutilized sugars	Check through chemical treatment methods, such as: (i) addition of chelating agents to remove divalent cations essential for phage infection, and (ii) use of non-ionic detergents and antibiotics

To this end, substantial efforts have been expended to develop a superior hyper-butanol-producing strain to revive industrial ABE fermentation, including: (1) expanding the selection of low-cost feedstock; (2) improving the butanol titer and tolerance; (3) adopting continuous or repeated fed-batch fermentations, and (4) increasing butanol selectivity with efficient recovery techniques (Table 1) [12, 13]. Meanwhile, to overcome the intrinsic weaknesses of clostridia, nonsolvent-producing species, such as *Escherichia coli* and *Saccharomyces cerevisiae*, have been engineered by synthetic biological means to produce butanol [14]. As a consequence, various review articles have been published recently that summarize the general aspects of ABE fermentation [1] and focus on the metabolic pathways and metabolic engineering tools of clostridia [15, 16], redox potential regulation [17], economical substrate-derived carbohydrates [18–20], butanol toxicity and tolerance [21, 22], butanol fermentation technology [23], as well as butanol separation techniques [24–26]. Here, the intention of this review is to sum up the development of clostridia-based ABE fermentation from strain improvement to bioprocess optimization, particularly focusing on recent research progresses in dissecting metabolic pathway and relevant regulation mechanism of butanol production in solventogenic clostridia. Additionally, the derivative strategies that were used to engineer clostridia to improve cellular performance and fermentation process are also discussed, providing guidelines for enhancing the overall performance of ABE fermentation, and converting ABE to the production of higher value-added chemicals.

## Microorganisms and metabolic characteristics of ABE fermentation

### Microorganisms for ABE fermentation

The strictly anaerobic genus *Clostridium* can select from at least 14 distinct families of glycosyl hydrolases, including α-amylase, α-glucosidase, β-amylase, β-glucosidase, glucoamylase, pullulanase, and amylopullulanase, to degrade carbohydrate polymers to economically produce biochemicals (such as butyrate, ethanol, butanol, and hydrogen) [27–29]. However, only four species, *Clostridium acetobutylicum*, *Clostridium beijerinckii*, *Clostridium saccaroperbutylacetonicum*, and *Clostridium saccharoacetobutylicum*, exhibit significant activity for synthesizing butanol under appropriate conditions [30, 31], among which *C. acetobutylicum* is the model organism for ABE fermentation, while *C. beijerinckii* is considered a potential candidate for lignocellulosic biomass-based ABE fermentation [32, 33].

As an alternative strategy, several well-characterized microorganisms (such as *E. coli* [34], *S. cerevisiae* [35], *Pseudomonas putida* [36], and *Bacillus subtilis*) could also be engineered to synthesize butanol, and have been attracting increasing interest of late. Compared with the native butanol-producing microbes, these well-characterized microorganisms have fast growth rates, well-characterized genetic backgrounds, and well-established genetic manipulation systems, and are associated with more economically viable large-scale production processes [37, 38]. More importantly, some engineered strains have a superior butanol yield and fewer by-products than clostridia, and are thus excellent hosts for butanol production [39].

### Metabolic characteristics of ABE fermentation

**Fig. 1:**
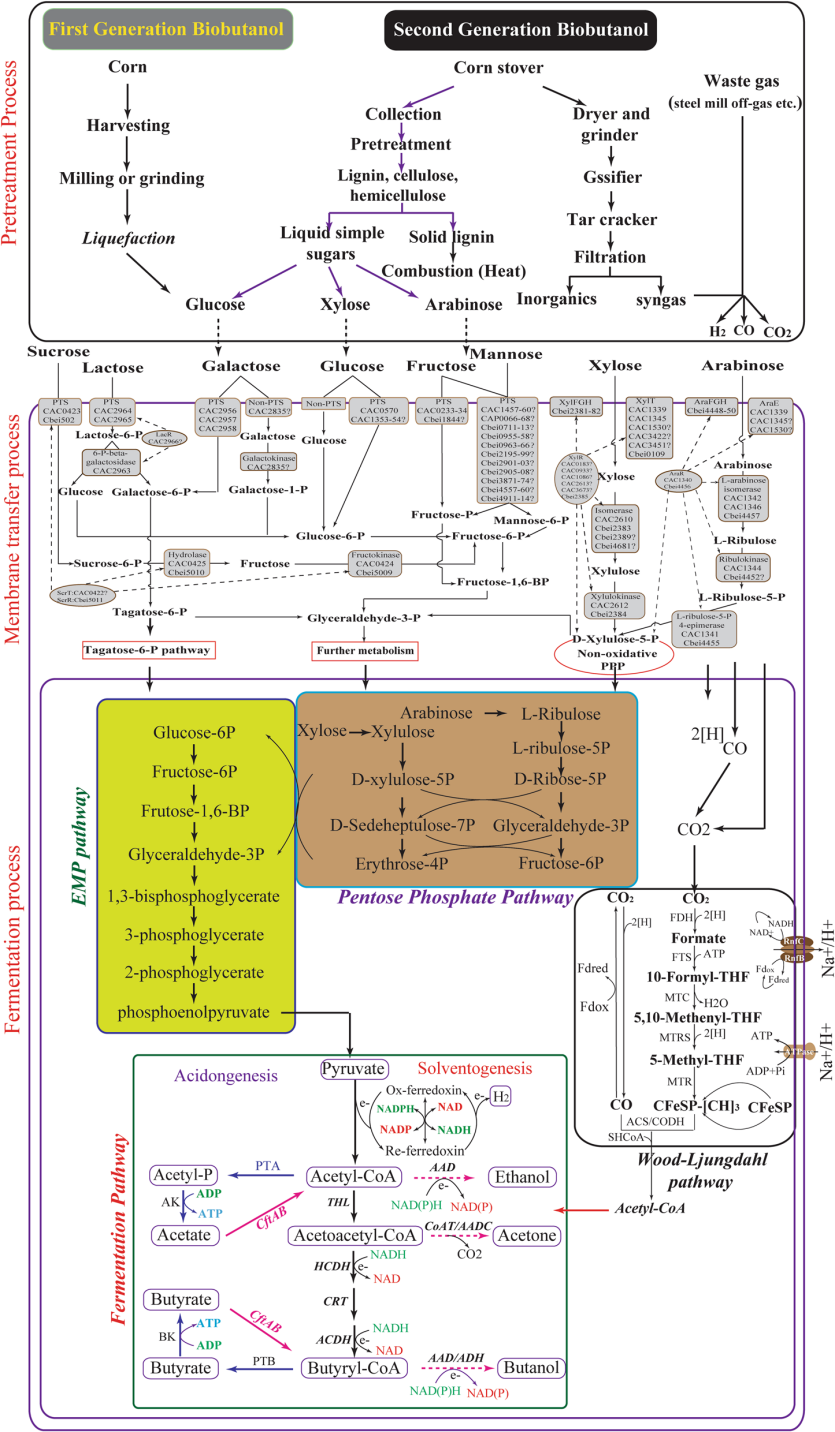
Simplified metabolism of biomass for microbial *n*-butanol fermentation by solventogenic clostridia through pretreatment process, membrane transfer process and fermentation process, especially depiction of metabolic pathways, energy conservation and the related enzymes for butanol production, in which glycolysis, the pentose phosphate pathway, and the Wood–Ljungdahl pathway are necessary for growth on hexoses, pentoses, and syngas, respectively. Abbreviations of the different metabolites or enzymes are as follows: *AAD* alcohol/aldehyde dehydrogenase, *AADC* acetoacetate decarboxylase, *ACDH* acyl-CoA dehydrogenase, *ACS/CODH* acetyl-CoA synthase/CO dehydrogenase, *ADH* alcohol dehydrogenase, *AK* acetate kinase, *ALDC* acetolactate decarboxylase, *ALDH* aldehyde dehydrogenase, *BK* butyrate kinase, *CAT* CoA transferase, *CFeSP* corrinoid iron–sulfur protein, *CRT* crotonase, *Etf* electron-transferring flavoprotein, *Fd* ferredoxin, *FDH* formate dehydrogenase, *FTS* formyl-THF synthase, *MTC* methenyl-THF cyclohydrolase, *MTD* methylene-THF dehydrogenase, *MTR* methyl transferase, *MTRS* methylene-THF reductase, *PFK-1* phosphofructokinase, *PFOR* pyruvate:ferredoxin oxidoreductase, *PGK* phosphoglycerate kinase, *PGM* phosphoglycerate mutase, *PK* pyruvate kinase, *PTA* phosphotransacetylase, *PTB* phosphotransbutyrylase, *THL* thiolase, *TPI* triosephosphate isomerase (adapted from [10, 29, 123])

Typically, various clostridia have similar metabolic pathways for ABE fermentation, which can be divided into three different growth phases (acidogenesis phase, solventogenesis phase, and sporogenesis phase) and produce three major kinds of products: (1) solvents (acetone, ethanol, and butanol); (2) organic acids (acetic acid and butyric acid), and (3) gases (carbon dioxide and hydrogen) [40, 41] (Fig. 1).

#### Acidogenesis and solventogenesis phases

During exponential growth, the acidogenesis phase commences and is accompanied by changes in the intracellular microenvironment due to the accumulation of acetate and butyrate. As a result, a surplus of adenosine triphosphate (ATP) is generated as much as possible under anaerobic conditions, but a balance between the formation and utilization of reducing equivalents [reduced/oxidized nicotinamide adenine dinucleotide (phosphate) ratio NAD(P)H/NAD(P)^+^] is unconditionally required for non-respiratory metabolism [42].

At the final stage of the acidogenesis phase, the severely acidified environment (pH 4.5) and the higher levels of ATP and NAD(P)H/NAD(P)^+^ become the primary stress signals for activating the synthesis of solventogenic enzymes, shifting the metabolic activity rapidly from the acidogenesis phase to the solventogenesis phase [15, 43]. Subsequently, the activation of gene circuitry could be triggered by various environmental stresses, such as butyryl-phosphate [44], formic acid [45], and other environmental factors [46], which would then alter cellular activities to produce solvents [47]. As a consequence, acetate and butyrate are re-assimilated to generate coenzyme A (CoA) derivatives (acetyl-CoA and butyryl-CoA), using acetoacetyl-CoA as the CoA donor, and ethanol and butanol are produced by different dehydrogenases under the limitation of reducing equivalents [15, 48] (Fig. 1).

#### Sporogenesis phase

After entering the stationary phase, the cells synthesize granulose as an intracellular storage compound, and then the sporulation process is initiated along with the cessation of solvent formation [49]. From the viewpoint of industrial applications, sporulation is an intrinsic drawback for clostridia, and a solvent-producing, non-sporulating strain may be more desirable for ABE fermentation, as avoiding sporulation without ceasing solvent formation would benefit specific cell productivity and simplify bioprocessing. However, the sporulation process is a carefully orchestrated cascade of events at both the transcriptional and posttranslational levels involving a multitude of sigma factors, transcription factors, proteases, and phosphatases, making the regulation mechanism of the sporulation process still incompletely understood at the molecular level [50, 51]. As for solventogenic clostridia, sequenced *Clostridium* genomes contain genes for all major sporulation-specific transcription and sigma factors (*spo0A*, *sigH*, *sigF*, *sigE*, *sigG*, and *sigK*) that orchestrate the sporulation program [52, 53]. More importantly, it is unclear how sporulation-specific sigma factors affect solvent formation [54], and even some asporogenous mutants are unable to synthesize solvents [7]. For example, inactivation of *б*^*F*^ in *C. acetobutylicum* could effectively block sporulation prior to asymmetric division, but had no effect on solvent formation [54]. However, unlike the *σ*^*F*^ and *σ*^*E*^  inactivation strains, the *SpoIIE* inactivation strain did not exhibit inoculum-dependent solvent formation and produced good levels of solvents from both exponential- and stationary-phase inoculation [55]. At present, there are three strategies that could be used to construct asporogenous strains: random mutagenesis, engineering specific sporulation stages, and starting with asporogenous, non-solventogenic strains (such as M5 and DG1) [56].

### Central metabolic pathway for butanol synthesis

With the publication of genomic data for several solventogenic clostridia, such as *C. saccharobutylicum* NCP262 [57], *C. beijerinckii* [58], *C. acetobutylicum* ATCC 824 [59–61], *C. acetobutylicum* DSM 1731 [62], and *C. acetobutylicum* EA2018 [63], the metabolic pathway for butanol production has been comprehensively elucidated and is mediated by CoA (Fig. 1) [64, 65].

#### Carbon metabolism

In brief, clostridia species can uptake a wide range of hexoses, pentoses, and oligomers through the phosphoenolpyruvate-dependent phosphotransferase system (PTS) and/or non-PTS transport systems [66, 67]. Subsequently, hexoses and pentoses [first via the pentose phosphate pathway (PPP)] are degraded to pyruvate through the Embden–Meyerhof–Parnas (EMP) pathway along with the production of ATP and NADH [68, 69]. Finally, the key intermediates of acetyl-CoA and butyryl-CoA are converted into oxidized products (i.e., acetone, acetate, or CO_2_) or reduced products (i.e., butanol, ethanol, or butyrate) via six key enzymes (thiolase, 3-hydroxybutyryl-CoA dehydrogenase, crotonase, butyryl-CoA dehydrogenase, butyraldehyde dehydrogenase, and butanol dehydrogenase) [70] (Fig. 1). Theoretically, 1 mol of glucose can be converted into 1 mol of butanol (0.41 g/g), 1 mol of acetone (0.32 g/g), or 2 mol of ethanol (0.51 g/g), but the actual ABE yield is significantly decreased due to the production of biomass, the non-assimilation of acetic acid and butyric acid, and the formation of other carbohydrates.

#### Redox metabolism

As shown in Fig. 1, synthesizing 1 mol of butanol consumes 4 mol of NADH, but the reduction in NADH level occurs during the phase transition, suggesting that a high NADH/NAD^+^ ratio benefits redistribution of the metabolic flux and solvent production [71, 72]. Therefore, increasing the intracellular NADH level (e.g., by up-regulating the NADH formation pathway or down-regulating the NADH consumption pathway) contributes to enhanced butanol formation [73]. In clostridia, three metabolic pathways regulate intracellular NADH/electron flow: (1) NADH-ferredoxin reductase and hydrogenase [74]; (2) Bcd/EtfAB complex and hydrogenase [75], and (3) bifurcating hydrogenase oxidizing NADH and ferredoxin simultaneously [76]. Recently, with the elucidation of the crystal structure of *C. acetobutylicum* thiolase (CaTHL) in reduced/oxidized states, CaTHL could be mediated by the redox-switch modulation through reversible disulfide bond formation between two catalytic cysteine residues, Cys88 and Cys378, and then affected overall metabolic flux distribution and the acidogenic to solventogenic phase transition, providing metabolic engineering and fermentation strategies for enhancing butanol production [77].

#### Energy metabolism

Similar to the dosage effect of NADH, high ATP levels not only effectively drive carbon flux towards butanol by blocking butyrate synthesis, but also improve cellular performance by alleviating butanol toxicity [78, 79]. However, the formation of butanol effectively inhibits ATP generation, and then down-regulates the expression of pyruvate kinases and phosphoglycerate kinase [80]. Considering both carbon and redox balances, 2 mol of ATP are produced by glycolysis and the reducing equivalents can be regenerated in the butanol pathway, and then the reduced ferredoxin transfers its electrons to NAD(P)^+^ with no molecular hydrogen formed. Therefore, artificially increasing the ATP accumulation (e.g., by inactivating the acetate and butyrate pathways) could improve the production and selectivity of butanol, but the energy distribution has become the challenge [14, 81]. More importantly, energy conservation in anaerobic bacteria is quite difficult and almost nothing is known about the genetic regulation of energy and redox status in clostridia.

Thus far, although the solvent-producing pathway has been comprehensively elucidated, differentiation of clostridia (i.e., sporulation) is still poorly understood. In particular, the regulatory mechanism of granulose formation and reutilization, the regulatory mechanism underlying the transition between acidogenesis and solventogenesis, and the molecular regulatory switches between sensing and transferring redox signals are still unknown [1, 82].

## Improvement in cellular performance for ABE fermentation

**Fig. 2:**
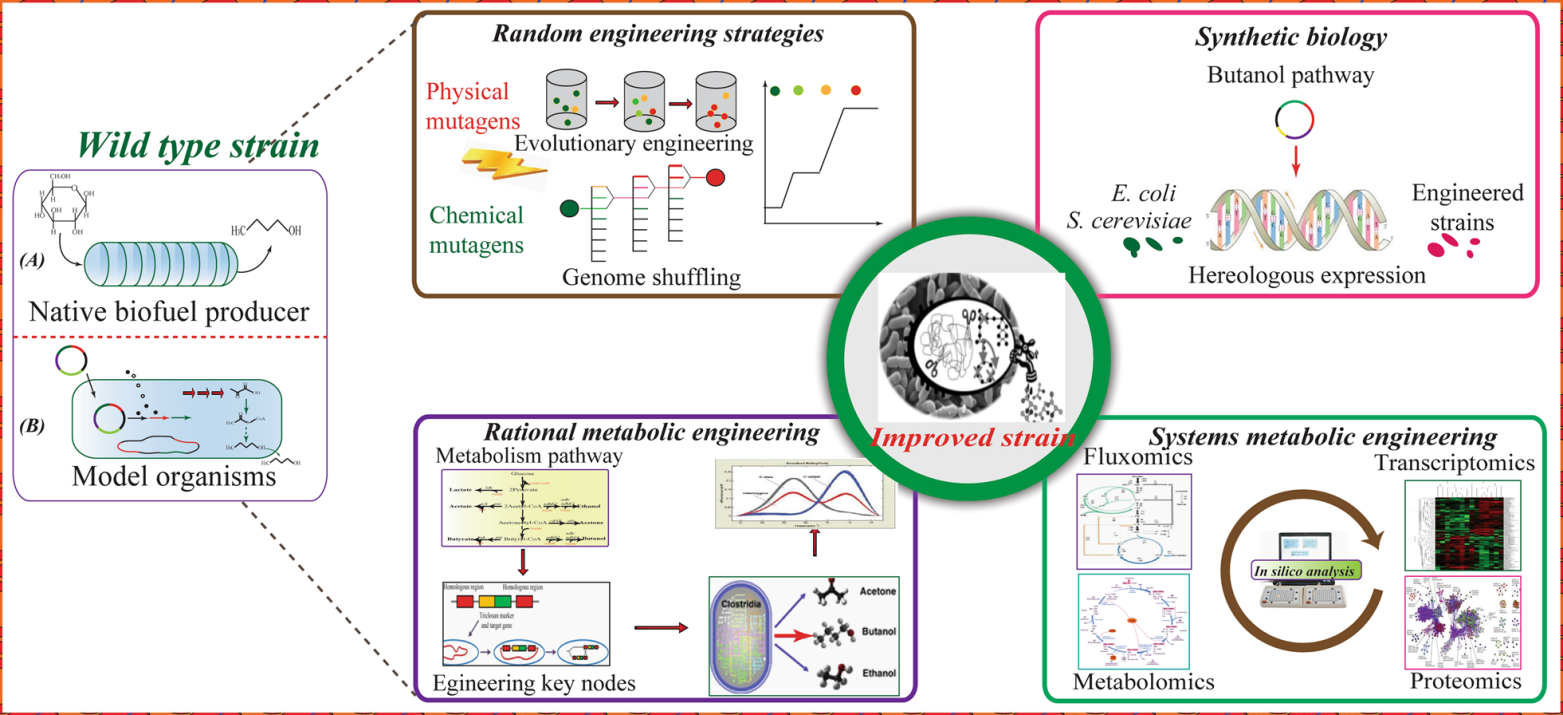
Strategies for improving cellular characteristics though random engineering, rational metabolic engineering, systems metabolic engineering, and synthetic biology (adapted from [222])

For ABE fermentation the biggest challenge is the low butanol yield due to the significant production of by-products and butanol toxicity. To this end, several phenotypic traits (such as solvent tolerance, aerotolerance [83], or abolished sporulation [84]) were chosen as important selective principles underlying the development of robust strains with superior metabolic capabilities [85] (Fig. 2).

### Strain improvement by mutation

During the pre-genomic era, mutagenesis of clostridia through physical or chemical methods was routinely and successfully executed to obtain the desired phenotypes (Table 2). For example, combining with the selective enrichment of glucose analogue 2-deoxyglucose and *N*-methyl-*N*-nitro-*N*-nitrosoguanidine (NTG) mutagenesis, a super butanol producing strain C. *beijerinckii* BA101 was obtained that significantly increased the butanol and total solvent yield from 9 g/L and 13 g/L to 19 g/L and 29 g/L, respectively [2]. Similarly, through butanol resistance screening and NTG mutagenesis, *C. acetobutylicum* EA2018 was also successfully isolated with a higher acetone–butanol–ethanol ratio of 2:7:1 (mass ratio) [63]. Furthermore, with a combination of chemical mutagenesis, genome shuffling, and butanol exposure, the mutant produced 20.1 g/L of butanol at 0.35 g/L/h, which were, respectively, 23.3% and 40.0% higher than the parent strain [86]. However, although traditional random mutagenesis combined with the screening method was still the most successful strategy for developing a high butanol-producing strain, it was difficult to further improve the physiological properties of the mutant due to the unknown and complex genotypic/phenotypic changes associated with identifying the modified genes [87].

**Table 2:** Application of various strategies to improve cellular performance for ABE fermentation

Strain	Strategy^a^	Physiological characteristics (control/engineered strain)	Refs.
Mutagenesis strategies			
*C. acetobutylicum BKM19*	Random mutagenesis, screening cells on fluoroacetate plates	Butanol: 15.9/17.6 g/L Total solvent: 24.9/32.5 g/L	[195]
*C. acetobutylicum* JB200	Evolution in a fibrous bed bioreactor	Butanol: 12.6/21.0 g/L Total solvents: 19.4/32.6 g/L	[93]
*C. acetobutylicum* GX01	NTG, genome shuffling and butanol exposure	Butanol: 16.3/20.1 g/L Total solvents: 26.4/32.6 g/L	[86]
Metabolic engineering strategies			
Enhancing butanol production			
*C. acetobutylicum* ATCC 824	Overexpressing both *pfkA* and *pykA* genes	Butanol: 14.78/19.12 g/L Total solvents: 21.76/28.02 g/L	[78]
*C. acetobutylicum* JB200	Disrupting the *cac3319* gene	Butanol: 12.6/18.2 g/L	[93]
*C. beijerinckii CC101*	Overexpressing *adh*E2 and *ctf*AB	Butanol: 2.6/12.0 g/L	[223]
Increasing butanol selectivity			
*C. acetobutylicum* ATCC 824	Knocking out *solR*	Butanol: 5.48/17.79 g/L Butanol ratio: 55%/66%	[224]
*C. acetobutylicum EA 2018*	Disrupting the acetone pathway by TargeTron technology	Butanol: 7.4/13.6 g/L Butanol ratio: 71/82%	[43]
Improving butanol tolerance			
*C. acetobutylicum*	Overexpressing HSP genes *grpE* and *htpG*	Improved butanol tolerance with increases of 25% (*grpE*) and 56% (*htpG*)	[167]
*C. acetobutylicum*	Overexpressing *gshAB* genes from *E. coli*	Increased cell resistance against butanol stress (from 14.5 to 18 g/L), and aero-stress	[83]

^a^*NTG N*-methyl-*N*-nitro-*N*-nitrosoguanidine

### Strain improvement by metabolic engineering

To address the drawbacks of mutagenesis in theory, various omics technologies (transcriptomics, proteomics, and metabolomics) have been applied to systematically elucidate the complex genotypic/phenotypic changes at different levels [88], providing an amount of targeted genes or proteins that further improve cellular performance by considering the metabolic pathway in its entirety [89].

#### Engineering tools for clostridia

**Fig. 3:**
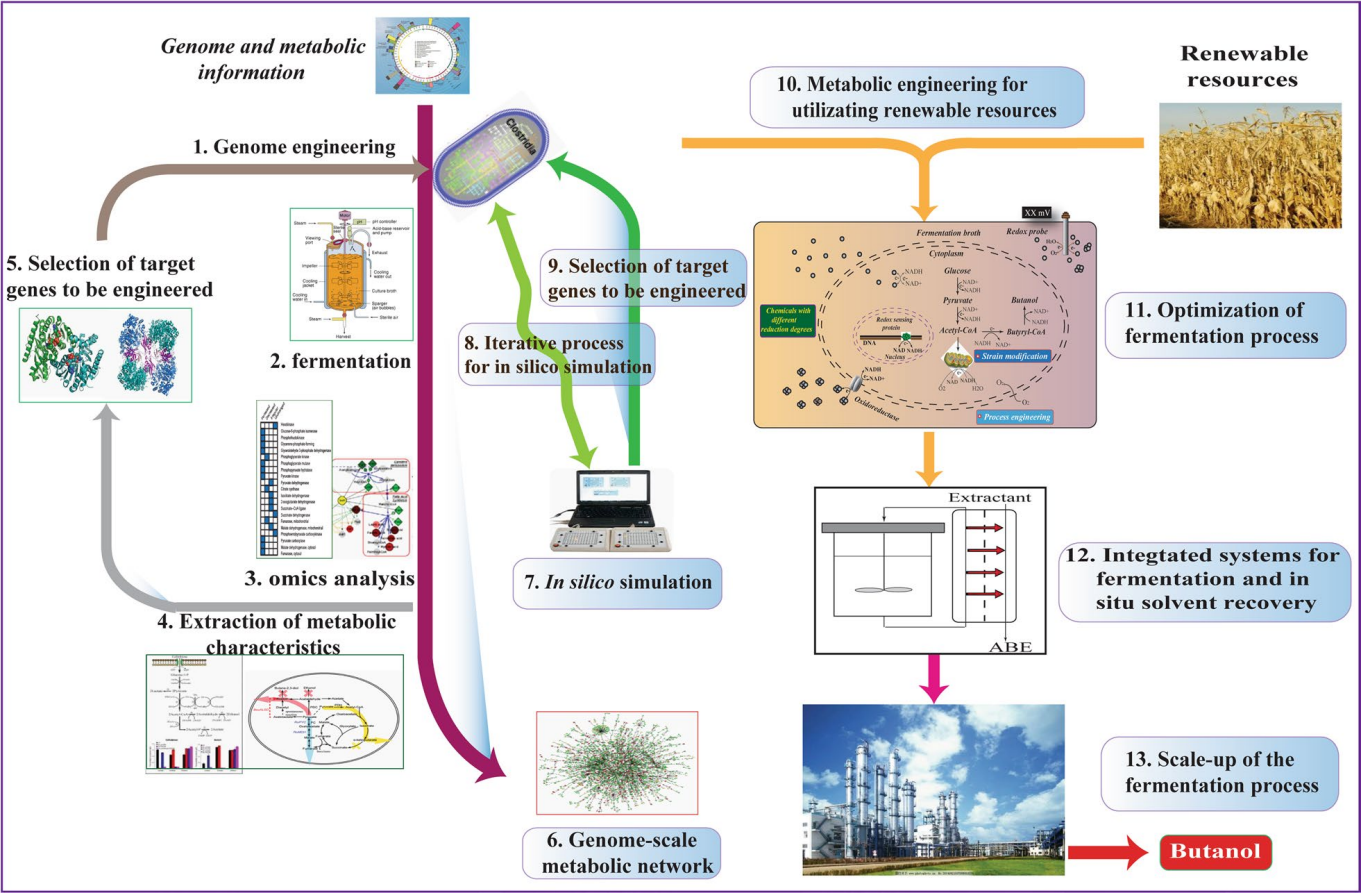
Strategies for developing an integrative bioprocess based on systems metabolic engineering for ABE fermentation (adapted from [203])

Compared to well-characterized microorganisms, *Clostridium* is notorious for the difficulty of genetic manipulations, and consequently only a few genetic tools can be used for engineering clostridia. With respect to gene cloning, much progress involving shuttle vectors, transformation and conjugation, circumvention of restriction barriers, and transposon mutagenesis, has been made in heterologous expression in clostridia [90] (Table 3). However, due to the low frequencies of transformation and recombination, as well as the double crossover integration, only three types of vectors, non-replicative and replicative integrative plasmids, a group II intron-based TargeTron technology and ACE (allele-coupled exchange) vector, have been developed for gene inactivation in clostridia [61, 91, 92]. Fortunately, the clustered regularly interspaced short palindromic repeats (CRISPR)/CRISPR-associated protein 9 (Cas9) system has been successfully applied to edit the genomes of *C. cellulolyticum* [93], *C. acetobutylicum* [4], *C. tyrobutyricum* [94], and *C. beijerinckii* [95], achieving multiple genome-engineered mutants. Furthermore, a synthetic small regulatory RNA (sRNA)‐based system, consisting of a target recognition site, MicC sRNA scaffold, and an RNA chaperone Hfq, was also developed to control or knock down gene expression for *C. acetobutylicum* with stability and efficiency [96]. More importantly, with the combination of high-throughput technologies, large amounts of omics data, and advances in computational biology, several genome-scale metabolic models (GSMMs) were constructed for clostridia [97, 98], providing the vital platform for visualizing the metabolic changes at a global level and predicting cellular phenotypes from genotype [99, 100] (Fig. 3).

**Table 3:** Operational tools of metabolic engineering for clostridia [101]

Engineering tool	Characteristics	Applications	Refs.
(i) Plasmids for gene overexpression			
pMTL80000 plasmid series	(1) A standardized modular *E. coli*/*Clostridium* shuttle vector system	Allowing reliable stringency and a broad range of inducibility	[225]
	(2) Uses native clostridial promoters or inducible promoters to promote plasmid-borne gene expression		[226]
*Bacillus*/*C. acetobutylicum* shuttle vector pFNK1	Harbors the replicon derived from pIM13 and a macrolide lincosamine resistance gene as selection marker	Overexpression of acetoacetate decarboxylase (*adc*) and phosphotransbutyrylase (*ptb*) genes in *C. acetobutylicum* ATCC 824	[227]
Plasmids pAN1 and pAN2	Contains compatible *E. coli* replicons and antibiotic resistance genes	Expressing the ϕ3TI methyltransferase gene from *B. subtilis* in *C. acetobutylicum*	[92]
Derivatives of pSOS94 and pHT3 plasmids	Gene expression reporter system using the *lacZ* gene from *Thermosulfurogenes* EM1	Actively expressing β-galactosidase and phosphotransbutyrylase in *C. acetobutylicum* ATCC 824	[228]
(ii) Chromosomal integration for gene overexpression			
Cargo technique	Manipulates chromosomal content to knockout the targeted gene and integrate large DNA fragments into the chromosome	(1) Engineering *C. acetobutylicum* to convert acetone into isopropanol (2) Engineering *C. acetobutylicum* to secrete various synthetic cellulosome components	[229, 230]
(iii) Synthetic untranslated regions for gene overexpression			
Synthetic untranslated regions	A short single-stranded 5′ untranslated region (UTR) sequence	Short single-stranded UTR decreased gene expression, but addition of a stem–loop at the 5′ end of mRNA increased the levels of mRNA and protein expression	[231]
(iv) Integrative plasmids for gene inactivation			
Non-replicative suicide plasmids	Homologous recombination into the respective genes	Inactivating *SolR* to improve solventogenic phenotype	[232]
Replicating plasmids	Integration into a specific gene to inactivate	Using replicating plasmids to inactivate the genes *spo0A, sigE*, and *sigG*	[233]
CRISPR–Cas9 system	Clustered regularly interspaced short palindromic repeats system	Deleting spo0A with an editing efficiency of 100% in *C. acetobutylicum*	[94]
(v) Antisense RNA for gene down-regulation			
Plasmid-encoded asRNA	Knocking-out or down-regulating targeted genes	Down-regulating *spoII*E to prolong and elevate solventogenesis and delay initiation of endospore formation	[234]
(vi) Small RNA system for gene down-regulation			
Small regulatory RNA (sRNA)‐based system	Consisting of a target recognition site, MicC sRNA scaffold, and an RNA chaperone Hfq	Knocking down the *pta* gene expression in strain PJC4BK to reduced acetic acid production	[96]
(vii) ClosTron system and related techniques for gene inactivation			
Group II introns	(1) Comprising a multi-domain intron-encoded protein that mediates sequence recognition and self-splicing (2) Engineered by including a retrotransposition-activated marker providing erythromycin resistance after intron insertion (3) Another variant, combining group II intron and homologous recombination	(1) Alternating use of erythromycin and thiamphenicol resistance genes for generating various gene knockout mutants including three double- and two triple-knockout strains (2) An alternative gene deletion method that is very elaborate and time-consuming because of successive transfers and large numbers of colonies that need to be screened to identify positive clones	[235, 236]
(viii) Allelic exchange mutagenesis			
Allele-coupled exchange (ACE)	Developed to knock out genes or integrate large DNA fragments into the genome with the selectable phenotype	Generating stable deletion mutants of *C. acetobutylicum*	[61, 237, 238]

#### Strain improvement by metabolic engineering

##### Enhancement of solvent production

At present, two strategies, regulating genes associated with solvent formation and altering the metabolic regulatory system, have been developed to further enhance solvent production in clostridia [101]. First, to directly reinforce the butanol biosynthesis pathway, four genes (*pta*, *ack*, *ptb*, and *buk*) involved in short-chain fatty acid synthesis were individually or combinatorially knocked out in *C. acetobutylicum* ATCC 824, resulting in a higher titer (16 g/L) of butanol being produced in the *pta/buk* double-knockout mutant. Meanwhile, with the integration of an overexpressing variant *adh*E1 gene, the production of butanol was further increased to 18.9 g/L [102].

Additionally, regulatory proteins, affecting a larger set of pathways, programs, or signal transduction, could also be engineered to achieve desirable phenotypes. As a transcriptional regulator, SolR can positively control sporulation and solvent production, so that the combination of inactivating SolR and overexpressing *aad* was used to improve cellular performance, increasing the production of butanol, acetone, and ethanol to 17.6 g/L, 8.2 g/L, and 2.2 g/L, respectively [103]. More importantly, other regulatory factors (such as SpoIIE, sigF, sigE, and sigG) have been identified as being involved with improving cell growth and solvent formation, showing that understanding the regulatory mechanism of the solventogenic shift is of great interest, but identification of the molecular triggering machinery still presents an obstacle [104].

##### Improvement of butanol selectivity

Acetone, the major by-product representing 30% of the total mass, is considered the most undesirable product due to its poor fuel properties [105], and therefore inhibiting acetone production by developing a high butanol proportion or butanol-only strain could effectively improve the economics of ABE fermentation [43, 106]. But interestingly, disrupting acetoacetate decarboxylase (encoding by *adc*) effectively inhibits acetone production, increasing the butanol mass ratio from 70 to 80%, but significantly decreases the butanol production from 13.6 to 7.4 g/L [43]. Therefore, eliminating or reducing the acetone flux was not sufficient to increase butanol production, and even decreased butanol production due to an increase in the accumulation of acid [43]. In addition, engineering aldehyde/alcohol dehydrogenase (AAD) was also used to enhance butanol selectivity in *C. acetobutylicum*, dramatically increasing the butanol/ethanol ratios (B/E ratios) to 17.47 and 15.91 g butanol/g ethanol for AAD_F716L_ and AAD_N655H_, respectively, which were 5.8- and 5.3-fold higher than the wild-type AAD [107].

Recently, real progress in raising the butanol ratio was achieved using strains M5 and DG1 (mutants without megaplasmid pSOL1). As shown in Table 2, when both *adh*E1 and *ctf*AB were co-expressed in M5, the mutant could produce 11.4 g/L of butanol with a butanol mass ratio of 0.84 [106]. Apart from M5 and DG1, a novel advance involving disrupting the *adc* gene was developed, in which the production of acetone decreased from 2.83 to 0.21 g/L but the production of butanol was still 12–13 g/L, through pH-controlled fermentation and the addition of methyl viologen, so that the butanol mass ratio increased to over 82% [43]. However, because of the relatively poor understanding of solventogenic clostridia, great challenges still remain in realizing a real butanol-only process through metabolic engineering, such as how to block branch pathways without generating undesired phenotypes (e.g., acids assimilation, deficient growth rate), how to drive the carbon flow specifically to butanol, and how to provide sufficient reducing force to support butanol formation [27, 108].

##### Improvement of carbohydrate utilization

As reported in the literature, substrate costs, which constitute approximately 60% of the total process cost [1], are the most important factor affecting the economic feasibility of ABE fermentation. Therefore, exploring low-cost substrates (such as energy-dense lignocellulosic biomass [13, 18, 109], oleaginous microalgae [110], and greenhouse gases [111]) is essential to improve the economic feasibility of ABE fermentation.

As the most abundant form of carbohydrate, lignocellulosic biomass (including forest residues, agricultural residues, and energy crops) is one of the best solutions for sustainable development of ABE fermentation [112]. However, clostridia cannot directly utilize cellulose or lignocellulosic biomass as a carbon source for butanol production [112]. Therefore, ABE fermentation from lignocellulosic materials needs to be improved through metabolic engineering of clostridia (overexpressing the heterologous minicellulosomes [113, 114]) and/or pretreatment techniques. As for cellulosic ABE fermentation, it could be summarized as involving: (1) material pretreatment (reviewed elsewhere in detail [115]); (2) enzymatic hydrolysis of cellulose to monosaccharides (hexose and pentose); (3) sugar fermentation to butanol, and (4) product recovery by distillation [116]. However, the efficiency of the concurrent uptake and metabolism of hexose and pentose is significantly impeded by glucose-mediated carbon catabolite repression [29, 117]. To this end, some strategies have been developed to simultaneously utilize mixed sugars in clostridia, as follows: (1) engineering specific membrane-bound transport systems [118], such as deletion of xylose repressor (e.g., CcpA) [82] or overexpression of xylose transporter [119, 120]; (2) constructing a xylose pathway [121]; and (3) improving a cellular tolerance towards inhibitors contained in lignocellulosic hydrolysates [119, 122, 123]. For example, after alkali pretreatment and enzymatic hydrolysis, *C. acetobutylicum* can produce 14.17 g/L of butanol with a yield of 0.22 g/g sugars in a fed-batch fermentation from sugarcane bagasse [124]. However, the high cost of pretreatment processing has become the key factor for the economic feasibility of cellulosic ABE fermentation [10].

Recently, consolidated bioprocessing (CBP) has attracted increasing attention because it can accomplish hydrolytic enzymes production, lignocellulose degradation and microbial fermentation in one single step. Therefore, metabolic construction, isolation of novel cellulolytic/hemicellulolytic and solventogenic bacteria, or construction of microbial co-cultures to achieve direct butanol production from lignocellulose offers a promising alternative [125]. For example, with the expression of indigenous xylanase, *Clostridium* sp. strain NJP7 could produce 2.06 g/L of butanol and 0.54 g/L of isopropanol from hemicellulose through the simultaneous saccharification and fermentation via consolidated bioprocessing [126]. Furthermore, a newly isolated *Thermoanaerobacterium* sp. M5 could directly produce butanol (1.17 g/L) from xylan through CBP at 55 °C. More importantly, with the establishment of co-cultivation system consisting of *Thermoanaerobacterium* sp. M5 and *C. acetobutylicum* NJ4, it could effectively enhance the butanol titer to 8.34 g/L from xylan through CBP [127]. However, the CBP technology is still in its infant stage, and microorganisms, microbial consortia and/or condition at industrial scale should be further improved to achieve a high-yield and low-cost CBP process [128, 129].

To thoroughly resolve the debate between food vs. fuel, algal biomass and synthesis gas (syngas) were also investigated for butanol production [130, 131], and have become increasingly attractive in both academic and industrial circles (reviewed elsewhere in detail [10]). After the enzymatic hydrolysis, *C. beijerinckii* could produce a higher butanol production (7.16 g/L) and yield (0.42 g/g consumed substrates) with butanol selectivity (0.85 of mass ratio) from brown seaweed [132]. More interestingly, with the overexpression of *adhE2* and *fnr*, *C. carboxidivorans* could produce ~ 18% more butanol (0.35 g/L) and ~ 22% more ethanol (2.44 g/L) than the wild type in the syngas fermentation [133].

### Strain improvement by enzyme engineering

To date, most of the work to improve ABE fermentation has focused on increasing the quantity of enzymes involved in butanol biosynthesis, but significant progress has not been obtained. To this end, modifying enzymes with higher activities and specificities by enzyme engineering was developed as an alternative strategy [134, 135]. For butanol biosynthesis, most of the key enzymes have been purified and biochemically characterized (reviewed in detail elsewhere [15]), but only a few studies on engineering enzymatic properties were reported to improve ABE fermentation. For example, when the cofactor specificity of AdhE1 was attenuated by a simple D-485-G amino acid substitution, both NADPH and NADH could be used as electron donors, and subsequently, the AdhE1 activity was enhanced to further improve the carbon flux from acetyl-CoA to butanol via butyryl-CoA [102]. Furthermore, the thiolase of *C. acetobutylicum* was specifically engineered to reduce sensitivity towards coenzyme A (CoA‐SH), significantly alleviating feedback inhibition through three amino acid substitutions (R133G, H156N, G222V) and, correspondingly, increasing the production of ethanol and butanol by 46% and 18%, respectively, although acetone production was similar to the vector control strain [136]. More importantly, compared to the wide-type *C. acetobutylicum* thiolase (CaTHL), the CaTHL^V77Q/N153Y/A286K^ mutant exhibited higher activity with more than threefold. As a result, when the *thl*_*Ca*_^V77Q/N153Y/A286K^ gene was overexpressed in the *thl*A-knockdown mutant, the production of butanol was increased to 7.4 g/L, which was higher than that (4.5 g/L) obtained with the *thl*A-knockdown mutant complemented with the *thl*_Ca_ [77].

In summary, due to difficulties in genetic manipulation and complex physiology, a comprehensive understanding of the genes, pathways, and metabolic and regulatory characteristics is still lacking, and only limited metabolic engineering strategies have been successful in improving clostridia, so that traditional random mutagenesis is still the most successful method [25]. Therefore, the combination of metabolic engineering and evolutionary engineering was applied to further improve the overall performance of ABE fermentation. For example, with the integration of the overexpression of *adhE1* and *ctfA*–*ctfB*–*adc* and adaptive laboratory evolution approach, butanol production in the engineered *C. cellulovorans* increased from less than 0.025 g/L to 3.47 g/L in consolidated bioprocessing with deshelled corn cobs [137].

## Improvement of cellular robustness for ABE fermentation

### Confronting different environmental stresses

During ABE fermentation, cell growth and butanol production can be significantly influenced by several inhibitory factors (such as product inhibition, substrate inhibition, dead cells, low water activity, nutrient deficiency, and O_2_ stress) [138], which could typically be classified as [139] acid stress, solvent stress, or synergistic multiple stresses.

#### Acid stress

Generally, solvent production through the re-assimilation of acetate and butyrate is a stress response to the acid inhibition of cells [140]. However, when the accumulation of acids is at a high level, the acidification of the cytoplasm or anion accumulation can dramatically inhibit cell growth and solvent production [141]. Therefore, improving cellular tolerance towards carboxylic acids (acetate and butyrate) could prolong cell growth to achieve a higher cell density and result in synthesis of more acids during the acidogenic stage [142, 143], and then increase butanol production at the solventogenic phase [144].

Furthermore, as for the cellulosic ABE fermentation, a number of associated compounds, such as salts, weak acids (i.e., acetic, formic, and levulinic), furan derivatives [i.e., hydroxymethylfurfural (HMF) and furfural], and phenolic compounds (i.e., *ρ*-coumaric acid, ferulic acid, hydroxybenzoic acid, vanillic acid, and syringaldehyde), were uncontrollably generated along with the pretreatment and hydrolysis processes of lignocellulosic biomass [145, 146]. More importantly, the inhibitory effect of these compounds could be strengthened together due to a synergistic effect, and then severely affect microbial fermentation by low levels of ferulic acids and phenolic compounds [3, 147, 148].

#### Solvent stress

Due to its lipophilicity, butanol is in general extremely toxic for microorganisms, but much less is known about the specific impacts of solvents on clostridia [149]. When clostridia are exposed to butanol, adverse changes in phospholipid and fatty acid compositions of the cell membrane are induced, and then the unsaturated-to-saturated fatty acids ratio (U/S) decreases and the specific interactions of lipids are diminished [150]. More importantly, once butanol (a noxious organic solvent) enters the cytoplasmic membrane, it short-circuits the bilayer and creates a coupled system where interdigitated and splayed phospholipids coexist. Thus, it significantly impacts the physicochemical characteristics of the cell membrane, including preferential solute transport, glucose uptake, membrane permeability, maintenance of the proton motive force, the intracellular ATP level, and the activities of intrinsic membrane proteins [139, 151].

#### Synergistic effect of multiple stresses

The synergistic effect involves two aspects: relatively constant concentrations of toxic metabolites under chemostatically or well-controlled fed-batch fermentation and time course concentration changes in the toxic metabolites or concentration gradients developed under batch fermentation [152]. Therefore, effects created by supplementing fermentations with different levels of acetate, butyrate, and butanol individually were significantly different from those that occur during ABE fermentation, making the experimental results less meaningful [139]. As a result, the highest butanol titer produced by clostridia reported in laboratory research was ~ 20 g/L (the maximum tolerance of *C. beijerinckii* was 1.96%) [153, 154], whereas only ~ 12 g/L butanol was produced under industrial conditions due to multiple, even simultaneous stresses [3].

In general, developing a strain with high butanol tolerance is the prerequisite for biobutanol production but, apparently, engineering strains with stress tolerance is more complicated than altering the butanol ratio and butanol production by metabolic engineering [22, 155, 156].

### Improvement of cellular robustness during ABE fermentation

**Fig. 4:**
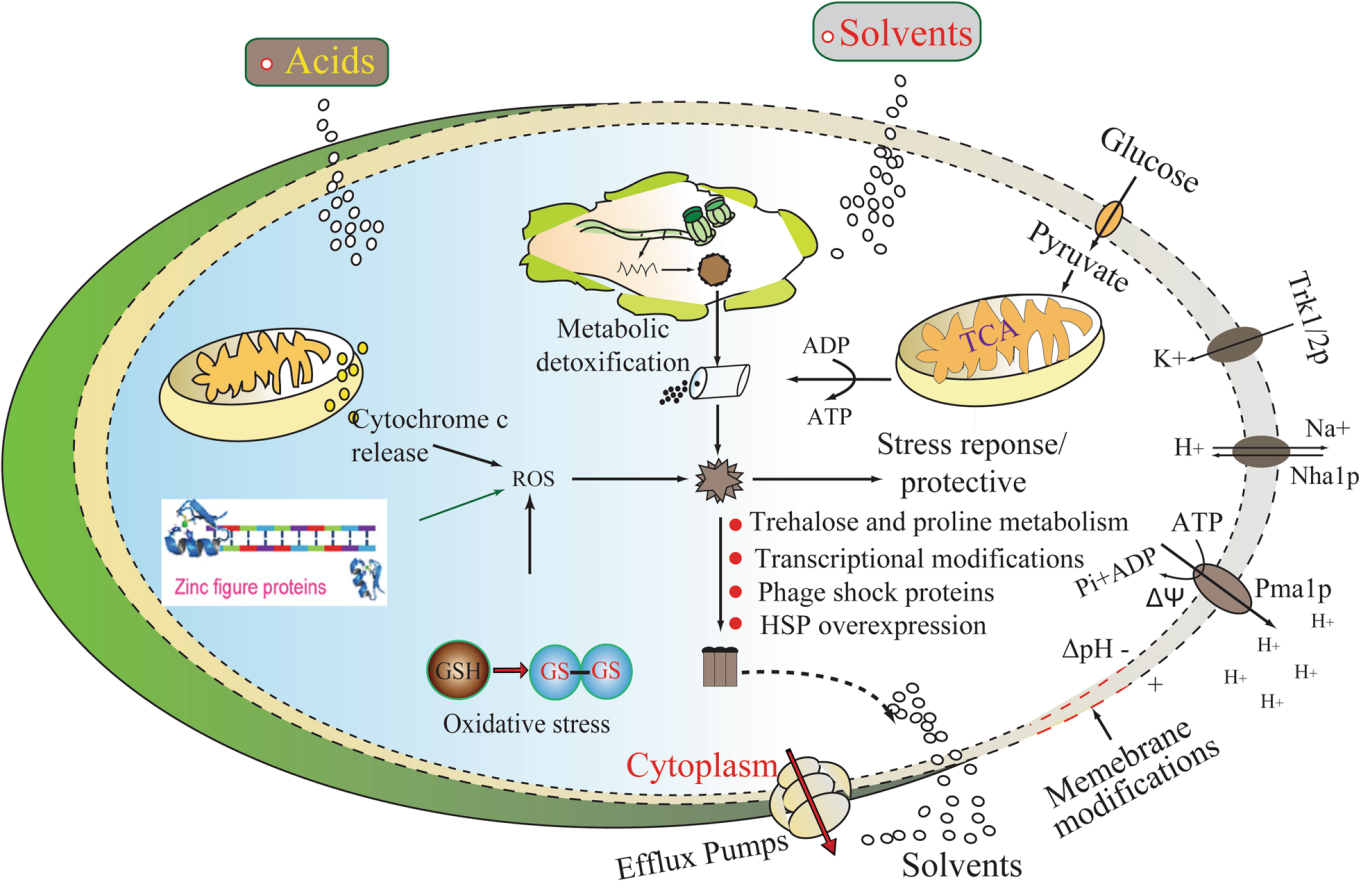
Schematic diagram showing a prototype of environmental tolerance mechanisms in clostridia

During the long evolutionary process, microorganisms automatically develop a series of strategies to improve cellular robustness against environmental stresses (Fig. 4), including: (1) metabolic detoxification; (2) heat shock stress proteins (HSP); (3) the proton motive force and associated energy production; (4) molecular efflux pumps; (5) changes in cell membrane composition and biophysics, and (6) other transcriptional responses [22, 157–159]. At present, several butanol-tolerant strains have been obtained through classical chemical mutagenesis, continuous culture, and serial enrichment procedures [21, 160]. Unfortunately, many tolerant strains did not correspondingly display an improved capacity to produce butanol. More importantly, the molecular mechanism underlying butanol tolerance is still not comprehensively understood, making it difficult to further improve clostridia [48, 161].

#### Engineering individual genes or enzymes

As an effective strategy, the *gsh*AB genes, coding for γ-glutamate-cysteine ligase and glutathione synthetase, could be explored to improve cellular robustness. The overexpression of *gsh*AB genes gave a more robust phenotype of *C. acetobutylicum* with butanol resistance and aerotolerance [83]. More importantly, with the combination of disrupting *adc* and overexpressing the *gsh*AB cassette, the cellular robustness and fermentation performance were correspondingly enhanced, so that the production of butanol increased from 5.17 to 14.86 g/L with the cost being acetone production, which decreased from 2.64 to 0.15 g/L [162]. Furthermore, knocking-out a histidine kinase (*cac 3319*) effectively improved the cellular performance of *C. acetobutylicum*, so that the butanol titer and productivity increased from 12.6 to 18.2 g/L and 0.20 to 0.38 g/L/h, respectively, due to the enhancement of butanol tolerance [93, 163]. Similarly, a mutation in the *adhE* gene (D494G) was the key factor for several tolerant strains, as re-introducing the *adhE* gene (D494G) effectively increased cellular tolerance against several primary alcohols, such as butanol, isobutanol, and ethanol [164].

#### Transcriptional regulators

As an alternative strategy, several molecular chaperones, including groES, dnaKJ, hsp18, and hsp90, have been identified and characterized as potential target genes to improve cellular tolerance [152, 165, 166] (Table 2). For example, with the overexpression of GroESL and DnaK, derived from the extremely radio-resistant bacterium *Deinococcus wulumuqiensis* R12, the recombinant strains [designated 824 (dnaK R12) and 824 (groESL R12)] had higher tolerances against various stresses (such as butanol, furfural, oxidation, and acid), and produced 13.0 g/L and 11.2 g/L of butanol with increases of 49.4% and 28.7% compared to the parent strain, respectively [165]. Moreover, a genomic library was constructed to enrich genes involving butanol tolerance under increased butanol stress conditions, and the most enriched gene (CAC1869) contributed to an array of tolerance mechanisms, in which overexpression significantly increased butanol tolerance by more than 80% [149].

However, it should be noted that overexpression of HSP genes (e.g., *grp*E and *htp*G) effectively improved cellular robustness, but also significantly impaired solvent production, indicating that butanol tolerance and production capabilities are not necessarily linked together [167]. More interestingly, some negative control factors against butanol resistance were also described in clostridia, in which two adjacent genes of unknown functions were characterized by the comparative proteomic analysis. Inactivation of either or both genes effectively decreased sensitivity towards butanol stress, but overexpression of both genes inhibited cell growth in 1% butanol [168].

## Improvement of fermentation processes for ABE fermentation

Apart from modifications at the microbial level, engineering the fermentation process itself is another effective strategy for alleviating butanol toxicity and enhancing butanol production. At present, various fermentation processes, which determine capital investment in the upstream and downstream processes, feedstock consumption, and energy requirements, have been developed to further improve the efficiency of ABE fermentation.

### Optimization of culture conditions

#### Effects of exogenous additives on ABE fermentation

According to the metabolic pathway and physiological characteristics, various organic acids (such as acetate, butyrate, amino acids, and lactic acid) could serve as alternative substrates for butanol production, and then maintain the robust expression of enzymes associated during the solventogenic and solventogenesis phase [169, 170]. Interestingly, when butyrate was the sole carbon source, only 0.2 g/L of butanol was produced, but the production of butanol significantly increased to 10 g/L when both butyric acid and glucose were present, suggesting that butyric acid may be an important factor triggering solvent production [171]. More importantly, after optimization of the glucose concentration, butyric acid addition, and C/N ratio, the amounts of butanol and ABE production were further increased to 17 g/L and 21.71 g/L, respectively, in the scale-up fermentation of *C. acetobutylicum* YM1 [172, 173]. Similarly, with the addition of 30 mM ammonium acetate, the fermentation time of *C. acetobutylicum* EA was shortened about 12 h, and the yield of butanol increased from 8.3 to 13 g/L [174].

Generally, based on the critical micelle concentration, surfactants could self-assemble and into micelles, and then relieve the butanol toxicity against microorganisms by entrapping the butanol into micelles. To the end, adding surfactant was used to significantly improve the performance of ABE fermentation. Butanol is coated with surfactant to slow down its toxicity to microorganisms, so as to enhance butanol production. For instance, with the addition of non-ionic surfactant [3% (v/v) L62], the amounts of butanol and total solvents produced correspondingly increased to 15.3 g/L and 21 g/L, which were, respectively, 43% (w/w) and 55% (w/w), higher than the control. More interestingly, when the surfactant was added at 9 h, the productivities of butanol and total solvent further increased to 0.31 g/L/h and 0.39 g/L/h from 0.13 g/L/h and 0.17 g/L/h, respectively [175]. Likewise, a combined zinc-supplemented/magnesium-starved fermentation medium could also effectively improve central carbon metabolism through multi-level modulation, e.g., up-regulation of glycolytic pathway, up-regulation in thiolase, butyraldehyde dehydrogenase and butanol dehydrogenase, and down-regulation in alcohol dehydrogenase, and then enhanced glucose utilization, reduced ethanol production and induced solventogenesis earlier, making the production of butanol increased from 11.83 to 19.18 g/L [176].

#### Mixed cultivation for ABE fermentation

To further enhance the economic feasibility of ABE fermentation, mixed cultures with different microorganisms were developed to: (1) enlarge the range of substrates [177]; (2) increase the availability of intermediates [178, 179], and (3) decrease the cost of maintaining strict anaerobic conditions [180]. For instance, to eliminate the costly enzymatic hydrolysis step, a mixed culture of *C. thermocellum* and *C. acetobutylicum* was used for ABE fermentation, in which 40 g/L cellulose was directly utilized to produce 5.8 g/L butanol [181]. Similarly, when a mixed culture of *C. acetobutylicum* and *B. subtilis* without anaerobic treatment was used to reduce the application of costly reducing agents, 14.9 g/L of butanol was produced, which was 21.1% higher than that from a pure culture of *C. acetobutylicum* [182]. However, the greater possibility of infection by a bacteriophage when the number of transfer/sub-culturing steps is increased has become the major drawback, which has restricted the industrial application of co-culture systems.

#### Regulating the oxidation–reduction potential (ORP) for ABE fermentation

As shown in Fig. 1, a high level of NADH enhances butanol production at the expense of reduced acetone formation, suggesting that manipulating the intracellular redox balance at the molecular level or of the fermentation process may be an effective pathway to drive more carbon flux and energy towards butanol production [183, 184].

##### Intracellular regulators for redox balance

For clostridia, the electron flow is primarily regulated at the ferredoxin/hydrogenase node, so that reducing hydrogenase activities could effectively inhibit molecular hydrogen formation, driving electron flow towards butanol due to the regeneration of the NAD(P)^+^ pool [185]. Therefore, overexpressing NADH-dependent *adh*E2 could effectively regulate the redox rebalance, and subsequently further improve butanol production [80]. Similarly, Rex, a redox-sensing protein and transcriptional regulator, could effectively regulate the expression of genes involved in butanol pathways against the intracellular NADH/NAD^+^ shift. As a result, a Rex-negative mutant produced greater amounts of ethanol and butanol with less hydrogen and acetone as by-products [186].

##### Bioprocess engineering

Compared to the tedious task of genetic modification, bioprocess engineering (such as adding an electron carrier to strengthen NADH synthesis or aerating with CO to repress hydrogenases) could be explored with an immediate impact on environmental and intracellular ORP [187]. To this end, some artificial electron carriers (such as methyl viologen and neutral red) were added to drive the carbon flow from acids to alcohols: adding 2 g/L of Na_2_SO_4_ (an electron receptor) significantly increased butanol production, which reached 12.96 g/L, 34.8% higher than that of the control [188]. Likewise, when a mixture of 85% N_2_ and 15% CO was sparged during the ABE fermentation, hydrogenase activity and electron transfer were effectively suppressed, but the cellular NAD(P)H pool was significantly increased, improving the production of butanol from 4.8 to 7.8 g/L [189]. More interestingly, when the ORP of ABE fermentation was regulated at − 290 mV, solvent production could be initiated earlier, increasing solvent productivity by 35%, but the butanol yield was only slightly increased compared with that without ORP control [190].

### Optimization of the fermentation process

#### High-cell-density fermentation

Compared to aerobic fermentations, fermentations with clostridia have excellent specific carbon fluxes [103], but suffer from low cell density [a maximum absorbance of around 10–11 at 600 nm (A_600_)] due to product inhibition, some unknown quorum-sensing mechanism, or unsuitable bioreactor operation [191–193]. To this end, various fermentation processes (e.g., immobilized cell, batch, fed-batch, and continuous) have been developed to realize a high-cell-density fermentation by alleviating substrate and product inhibitions [33, 93] (Table 4).

**Table 4:** Comparison of different fermentation processes for butanol production

Fermentation process	Strain	Substrates	Yield (g/g)	Productivity (g/L/h)	Titer of ABE (g/L)	Refs.
Batch fermentation	*C. beijerinckii* P260	Barley straw	0.43	0.39	26.46	[239]
	*C. beijerinckii* BA101	Corn fibers	0.36–0.39	0.10	9.3	[33]
Fed-batch fermentation	*C. beijerinckii* P260	Wheat straw	–	0.36	16.59	[240]
	*C. saccharoperbutylacetonicum* N1–4	Synthetic medium with butyric acid	0.49	0.42	16.0	[241]
Continuous fermentation						
(i) Free cell continuous fermentation	*C. saccharobutylicum* DSM 13864	Sago starch	0.29	0.85	9.1	[242]
	*C. beijerinckii* BA101	Degermed corn	–	0.29–0.30	14.28	[194]
(ii) Immobilized cells continuous fermentation	*C. acetobutylicum* ATCC 55025	Corn	0.42	4.6	12.5	[243]
	*C. beijerinckii* BA101	Synthetic medium	0.36	12.43	8.8	[244]
(iii) Cell recycling and bleeding	*C. saccharoperbutylacetonicum* N14	Synthetic medium	–	11.0	8.58	[245]
	*C. acetobutylicum* BKM19	Clostridial growth medium (CGM)	0.34	21.1	23.5	[195]
In situ product recovery						
(i) Adsorption process						
Fed-batch fermentation	*C. acetobutylicum* ATCC 824	Glucose	0.22	0.45	54.6	[246]
(ii) Pervaporation process						
Batch fermentation	*C. acetobutylicum* ATCC 824	Glucose	0.38	0.76	201.8	[247]
(iii) Gas stripping process						
Batch fermentation	*C. acetobutylicum* JB200	Cassava bagasse hydrolysate	0.23	0.32	108.5	[248]
Batch fermentation	*C. beijerinckii* BA101	Glucose	0.47	0.61	75.9	[249]
Fed-batch fermentation	*C. beijerinckii* BA101	Glucose	0.47	1.16	233	[138]

Compared to fed-batch and batch fermentations (characterized by product inhibition and considerable down time) and despite a few disadvantages (such as high capital cost, phage contamination, and flocculation of bacterial growth), continuous processes (using free cells, immobilized cells, and cell recycling) offer a more attractive and productive alternative for commercial industrial ABE production. The various advantages include reductions in sterilization and re-inoculation times, superior productivity, and fewer substrate and butanol inhibitions [194]. For example, with the help of membrane cell-recycle bioreactor, a high cell density continuous ABE fermentation of *C. acetobutylicum* BKM19 was carried out to produce butanol and ABE with the volumetric productivities of 10.7 and 21.1 g/L/h, the production of 11.9 and 23.5 g/L, and the yields of 0.17 and 0.34 g/g, respectively, under the optimal operational condition [195]. Generally, a productivity of > 10 g/L/h, titer of > 10 g/L butanol, and yield up to 0.44 g/g could be achieved from a high-cell-density fermentation, a great success in ABE fermentation by *Clostridium* [196, 197].

#### In situ product recovery (ISPR) techniques

To further alleviate butanol toxicity, several in situ product recovery (ISPR) techniques, including pervaporation, adsorption, liquid–liquid extraction, and gas stripping, were also developed to integrate with the fermentation process for higher butanol productivity [198–202] (Table 5). For example, with the combination of fed-batch fermentation and gas stripping, the production, productivity, and yield of total solvent significantly increased to 233 g, 1.16 g/L/h, and 0.47 g/g, respectively [203]. However, there are several advantages and disadvantages of each recovery system for butanol production (Table 5). Reverse osmosis seemed to be the most preferable recovery technique from an economic point of view, but it is prone to membrane clogging or fouling [204]. Recently, a series of novel composite membranes (such as FAS cross-linked PDMS [205], PDMS-based pervaporation membranes [206], modified grapheme oxide with ionic liquid [207], and mixed matrix membranes [208]) were fabricated and applied to ISPR techniques, and displayed a more stable performance during long-time continuous operation of ABE fermentation [24]. More importantly, an ideal integrated recovery process should minimize energy consumption and concentrate butanol with high selectivity, so hybrid integrated recovery processes were also developed to compensate for the respective disadvantages of individual processes, and showed good potential for industrial ABE fermentation [191, 209].

**Table 5:** Advantages and disadvantages of in situ product recovery technologies

Technique	Advantages	Disadvantages	Possible solution	Refs.
Pervaporation	High selectivity, not harmful to microorganisms, energy efficient, no loss of fermentation broth and nutrients	Potential membrane leakage, possible membrane fouling, membrane material cost	Use a larger membrane surface	[146]
Liquid extraction	High selectivity, energy efficient, ease of implementation	Emulsion formation, toxicity of the extractant to microorganisms, extractant loss and nutrient loss, high price of ionic liquids	Exploit the more excellent extractants, such as those that are cost-effective, nontoxic, and have a higher partition coefficient and thermal stability	[85, 146, 250]
Gas stripping	Energy efficient, low consumption of water, no fouling, easy to operate, no harm to the culture	Circulation of anaerobic carrier gas, low selectivity, low efficiency	Develop two-stage gas stripping to further increase the ABE yield and the selectivity	[203, 248]
Adsorption	Energy efficient, easy to implement, relatively high biocompatibility	High price of the adsorbent, physical instability of the adsorbent, low selectivity, adsorbent regeneration	Adsorbent needs to be used for a large number of cycles to make it cost-effective and efficient	[221, 251, 252]
Perstraction	High selectivity, low toxicity to the culture	Fouling problem, emulsion formation, and material cost	Hampered by the high cost of membranes and the lower permeate fluxes	[221, 253]

## Engineering clostridia for other high value-added products

**Fig. 5:**
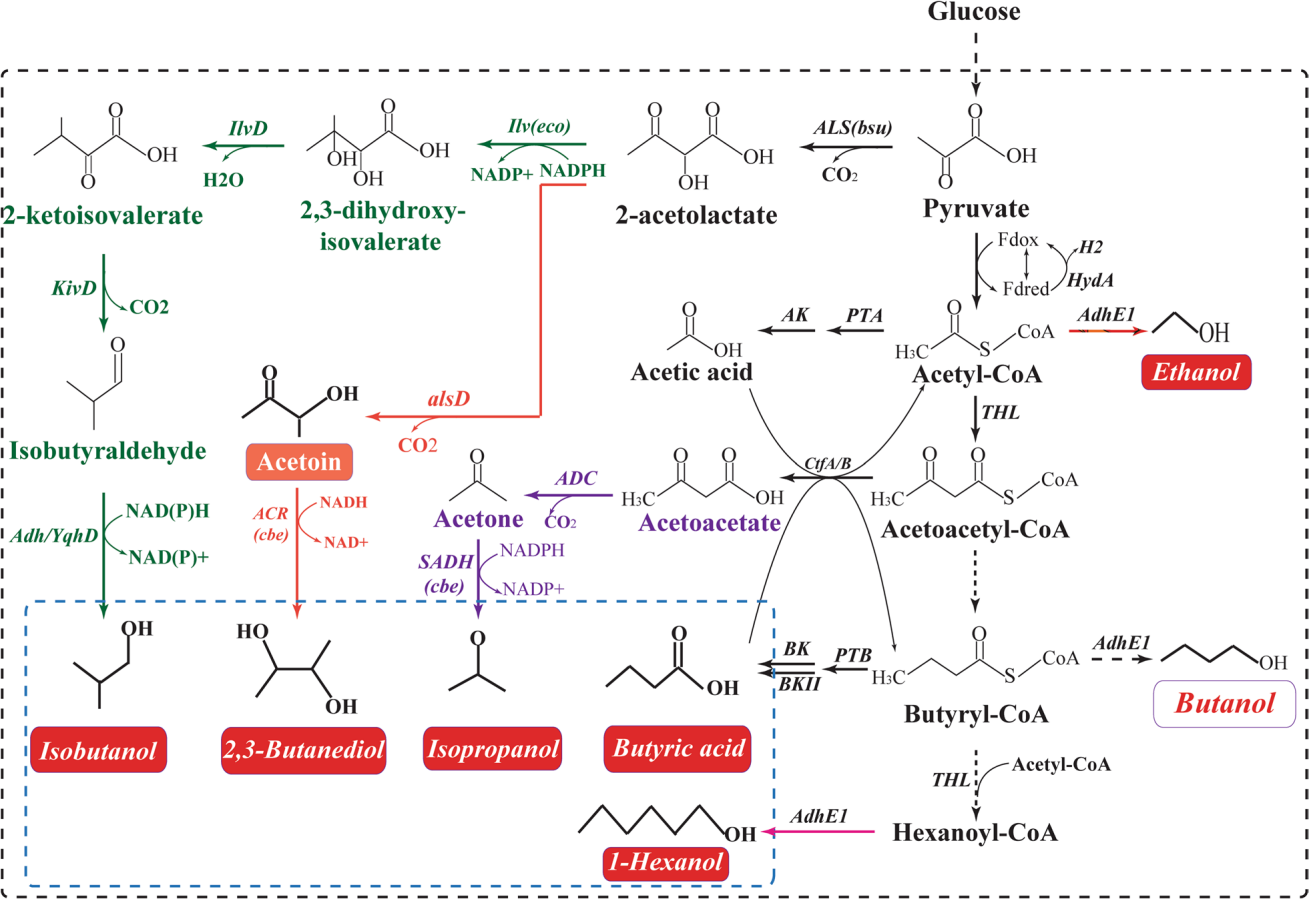
Metabolic engineering of clostridia (e.g., *C. acetobutylicum*) for: (1) the highly selective production of butyric acid; (2) the production of isopropanol, in which ADC and SADH are the native acetoacetate decarboxylase and secondary alcohol dehydrogenase from *C. beijerinckii*, respectively; (3) the production of other chemicals, such as ethanol, isobutanol, acetoin, 2,3-butanediol, and hydrogen (adapted from [215])

Acetone, the least attractive by-product, can be reduced to isopropanol through isopropanol–butanol–ethanol (IBE) fermentation, providing an ideal platform for direct utilization without prior separation of the solvents [210]. As shown in Fig. 5, with a combination of introducing the *sadh* gene and overexpressing the *ctfA*, *ctfB*, and *adc* genes, *C. acetobutylicum* could be engineered to be a typical IBE producer, so that 24.4 g/L IBE with a yield of 0.35 g/g glucose was produced by batch fermentation [211]. More importantly, when the genes *sadh* and *hydG* (putative electron transfer protein) were introduced into the hyper-ABE-producing *C. acetobutylicum* BKM19, 27.9 g/L of IBE with a yield of 0.29 g/g glucose were produced even without engineering the acetone pathway [212].

Additionally, *C. tyrobutyricum*, *C. butyricum*, *C. acetobutylicum*, and *C. thermobutyricum* could also be engineered as cell factories for producing butyric acid [213, 214]. When the *buk*, *pta*, and *ctfB* genes were knocked out in *C. acetobutylicum*, the butyric acid selectivity (BA/AA ratio) significantly increased to 14.3 g/g, but the production of butyric acid was only 23.8 g/L [91]. On this basis, a higher butyric acid selectivity could be obtained by increasing the NADH-driving force, as the pta–ctfB–buk–adhE1–hydA-deficient strain could produce 32.5 g/L of butyric acid with a yield of 0.41 g/g, a productivity of 0.89 g/L/h, and a BA/AA ratio of 31.3 g/g [91]. More importantly, with the development of interdisciplinary processes, the sole product or even the mixture produced by the ABE fermentation process can be further converted into high value-added products, such as 2,3-butanediol, short-chain esters, higher-molecular mass alkanes, riboflavin, and hydrogen, through enzymatic or chemical catalysis [215, 216] (Fig. 5).

## Conclusions and future prospects

An important bulk chemical, butanol has been attracting increasing interest from academic and industrial researchers. Over the past 100 years, great progress in strain improvement has been made through systems metabolic engineering integrated with process optimization [191]. However, ABE fermentation is an extraordinarily complex metabolic process with a large network of metabolic reactions as well as an associated gene regulatory program and environmental cues. Little is known about how the metabolic shift from acid to solvent production is regulated on the molecular level, making identification of the inducing signals, how many regulators are involved, and how regulator interactions are connected major topics for research [217–219]. Unfortunately, ABE fermentation is still not economically viable due to the high cost of feedstock, low butanol titer, and butanol toxicity [220, 221], and as butanol production still remains at low levels (< 21 g/L) and cannot compete with ethanol production (100 g/L) in batch fermentation mode [216].

In the future, to further improve the economic feasibility of ABE fermentation, multidirectional endeavors should probably continue to depend on a three-pronged approach that involves the upstream (strain development), midstream (innovative and advanced fermentation strategies), and downstream (in situ recovery and other cost-effective recovery) processes:

- Considering the intrinsic complexity of the metabolic network, targeting global regulators, transcription factors, and chaperones to further improve cellular performance (i.e., superior butanol tolerance, abandonment of sporulation, utilization of inexpensive carbon substrate, a single product (butanol) and higher butanol titer) at the global level.
- Constructing an excellent non-natural host to perfectly address the issues of low butanol tolerance and butanol yield, and poor utilization of low-cost substrates by integrating systems biology and synthetic biology.
- Developing and optimizing the butanol bioprocess by integrating fermentation and downstream processes, especially coupling with in situ methods for solvent extraction and recovery processes, such as gas stripping, advanced membrane separation with super critical extraction.
- Comprehensive application of fermentation by-products (large wastewater streams, cell mass, CO_2_, and H_2_) or in situ conversion of ABE into more value-added products through biological or chemical catalysis.

## Data Availability

Not applicable.

## Abbreviations

ABE: Acetone–butanol–ethanol
AAD: Aldehyde/alcohol dehydrogenase
ATP: Adenosine triphosphate
CoA: Coenzyme A
CRISPR: Clustered regularly interspaced short palindromic repeats
EMP: Embden–Meyerhof–Parnas
GSMMs: Genome-scale metabolic models
HSP: Heat shock stress proteins
IBE: Isopropanol–butanol–ethanol
NAD(P)H/NAD(P)^+^: Reduced/oxidized nicotinamide adenine dinucleotide (phosphate) ratio
ORP: Oxidation–reduction potential
PTS: Phosphotransferase system
PPP: Pentose phosphate pathway
U/S: Unsaturated-to-saturated fatty acids ratio
